# Fish By-Products: A Source of Enzymes to Generate Circular Bioactive Hydrolysates

**DOI:** 10.3390/molecules28031155

**Published:** 2023-01-24

**Authors:** Sandra Borges, Joana Odila, Glenise Voss, Rui Martins, Ana Rosa, José António Couto, André Almeida, Manuela Pintado

**Affiliations:** 1 Universidade Católica Portuguesa, CBQF—Centro de Biotecnologia e Química Fina—Laboratório Associado, Escola Superior de Biotecnologia, Rua Diogo Botelho 1327, 4169-005 Porto, Portugal; 2 ETSA, Empresa Transformadora de Subprodutos, 2660-119 Loures, Portugal

**Keywords:** viscera, protein hydrolysis, bioactive peptides, technological properties

## Abstract

Fish viscera are usually discarded as waste, causing environmental problems, or as low-value by-products. This study describes a self-sufficient and zero waste approach to obtain enzymes and protein hydrolysates from fish by-products. Firstly, recovery steps of viscera enzymatic extract were applied, and the resulting raw extract was stable at a pH range of 8–9 and at temperatures between 40 and 50 °C. The application of the extracted enzymes and alcalase on fish by-products hydrolysis was also determined. The selected conditions for the enzymatic hydrolysis were 10% (E/S) for 6 h using viscera enzymatic extract and 3% (E/S) for 2 h using alcalase. Fish protein hydrolysates (FPH) proved to have a notable antioxidant capacity with similar activity, ~11 mg ascorbic acid/g dry extract (ABTS assay) and ~150 mg Trolox/g dry extract (ORAC assay). FPH were also able to inhibit the angiotensin-converting enzyme, however, alcalase hydrolysates revealed a higher antihypertensive potential, IC_50_ of 101 µg of protein/mL. In general, FPH obtained by both enzymes systems maintained these bioactivities after the passage throughout a simulated gastrointestinal tract. The hydrolysates also displayed important technological properties, namely oil absorption capacity (~1 g oil/g sample) and emulsifying property (~40%). Therefore, it will be conceivable to use fish by-products based on a circular economy approach to generate added value compounds for animal and human nutrition.

## 1. Introduction

Fish production increases at an average annual rate of 3.2%, wherein the volume of global fish production amounted to 174.6 million metric tons in 2020. This fact, along with the growth in per capita fish consumption from 9.0 kg in 1961 to 20.2 kg in 2015, has generated large amounts of organic waste [1,2]. This waste include viscera, carcasses, heads, skin and bones, and represents more than 60% of biomass [3,4]. The biomass of fish viscera is usually discarded as waste or as low value by-products, which generate additional waste disposal and consequently environmental problems. Such fish by-products are conventionally employed to produce fishmeal and fertilizer; however, they are a potential source of high-value-added components when appropriate processes are applied, namely bioactive peptides, enzymes, fatty acids, water-soluble minerals, or biopolymers. Therefore, the marine bioprocess industry has a prominent potential to convert and apply these by-products as valuable products [5].

Proteases are one of the most important enzymes used worldwide, accounting for approximately 50% of the industrial enzymes market. These enzymes have several industrial applications in food, feed, agriculture, cosmetics and pharmaceutics [6]. Fish internal organs constitute approximately 20% of the marine biomass, among which fish viscera constitute a rich source of proteases, i.e., different digestive enzymes (pepsin, trypsin, chymotrypsin, elastase). The fish viscera comprise digestive tissues, namely stomachs, pyloric caeca, intestines, liver, pancreas, spleen and gonads [7].

Enzymatic hydrolysis has gained attention to obtain hydrolysates with improved and more tailored nutritional, technological and bioactive properties. Enzymatic processes have been implemented in a broad range of industries in recent decades because they are specific, fast in action and often save raw materials, energy, chemicals and/or water compared to conventional processes, such as acid or alkaline hydrolysis [8]. In food industries, enzymes are used to improve the flavour and to increase the protein yield of meat, poultry, fish and vegetables. Some research studies have been carried out with commercial enzymes, namely alcalase, aiming to obtain bioactive peptides from different fish varieties [9,10,11].

Fish is a source of protein that contains a high content, ranging 10–25%. Bioactive peptides produced from fish sources have gained interest in food and pharmaceutical areas. Bioactive fish protein hydrolysates (FPH) are desirable food ingredients due to their availability, reasonably low-cost extraction methods and their capability to have beneficial effects by displaying antioxidant and anti-hypertensive properties [12].

In normal conditions, an endogenous antioxidative defence system has the capacity to eliminate free radicals itself, maintaining a redox homeostasis. However, a disproportionate generation of these radicals can occur with an alteration in the redox homeostasis. This originates cellular damage, which can causes diseases such as atherosclerosis, arthritis, diabetes, and cancer [7]. Synthetic antioxidants agents such as butylated hydroxyanisole (BHA), butylated hydroxytoluene (BHT), tert-Butylhydroquinone (TBHQ), and propyl gallate (PG) are usually incorporated in food systems. However, because of their side-effects on human health, namely the induction of DNA damage and toxicity, the application of these substances as food additives is not favoured [13,14]. Therefore, there is a growing interest in finding antioxidants from natural resources, namely fish sources [15,16,17].

Hypertension is a major risk factor for different cardiovascular diseases. Angiotensin-converting enzyme (ACE) plays an important role in regulating blood pressure by converting angiotensin I to the vasoconstrictor angiotensin II, inactivating the vasodilator bradykinin. Therefore, ACE inhibition has been appointed as a strategy to treat hypertension [10]. Various studies have found ACE inhibitor peptides from fish by-products [10,18].

Therefore, the present work intends to apply a circular economy approach by obtaining an enzymatic extract from fish viscera to hydrolyse fish by-products and compare efficiency with a commercial alcalase. The effects of enzymes on physicochemical, technological and bioactive properties of FPH were also explored. In addition, simulated in vitro gastrointestinal digestion was conducted to investigate the impact on the antioxidant and anti-hypertensive properties of the derived products.

This will establish a circular and integral approach to valorise fish by-products, obtaining an enzymatic extract to be applied on the same by-products to obtain an added value functional ingredient rich in bioactive peptides. This will allow the increase of the current value of fish by-products (fish meal) towards applications for human nutrition and petfood, and will make the valorisation more circular and self-sufficient.

## 2. Results and Discussion

### 2.1. Composition Analysis of Fish By-Products and Viscera

Knowledge on the chemical composition of fish by-products is crucial for establishing their potential to be valorised in value added ingredients at a commercial and industrial level. The composition of fish by-products (meat and bones) and viscera (Table 1) demonstrated a high protein content and viscera also exhibited a high fat content. These data are within those found for fish, because generally fish contain 70–84% water, 15–24% protein, 0.1–22% fat, 1–2% minerals and 0.1–1% carbohydrate [19].

Each component can have different possible applications. Due to the high protein content, it is possible to produce protein hydrolysates and bioactive peptides. Therefore, it can be used as a dietary supplement with the high potential to reduce the malnutrition condition and simultaneously have beneficial health effects. Fish fat is well recognized for containing omega-3 fatty acids, docosahexaenoic acid (DHA) and eicosapentaenoic acid (EPA) that may play an extremely important role in cardiovascular diseases prevention. Hydroxyapatite can be obtained from fish minerals and can be used as a bone replacement or even in wastewater treatment.

### 2.2. Production of Enzymatic Extract from Fish Viscera

Enzymes from fish viscera were extracted by the procedure described below. The effect of pH and temperature in enzyme activity was determined using azocasein as a substrate [20] (Table 2). The proteolytic activity was studied over a pH range of 3.0–9.0 and it was determined that the optimum activity pH values of the viscera enzymatic extract are alkaline (pH values of 8 and 9). The optimum pH between 8 and 10 has been reported for enzyme activities of different fish species such as sardine (*Sardina pilchardus*) [21], zebra blenny (*Salaria basilisca*) [22] and bogue (*Boops boops*) [23], among others. The optimal temperature was evaluated, incubating the enzymatic extract for 4 h at a temperature range 40–60 °C and the enzymatic extract was revealed to be more stable at temperatures of 40 and 50 °C, having a loss of proteolytic activity at 60 °C. These results are similar to enzymes extracted from the pyloric ceca of jacopever (*Sebastes schlegelii*) and elkhorn sculpin (*Alcichthys alcicornis*), whose activities quicky fell above 50 °C [24].

Further studies should be performed to obtain a partial purification of the viscera enzymatic extract. Once a crude enzymatic extract is recovered by precipitation, it could be purified using chromatographic methods which include ion exchange, gel filtration, hydrophobic interaction or affinity [21]. Therefore, it will be important to develop a fast, efficient, economical and scalable approach for the separation and purification of enzymes, in order to enable their use in the food industry.

### 2.3. Enzymatic Hydrolysis of Fish By-Products

**Figure 1:**
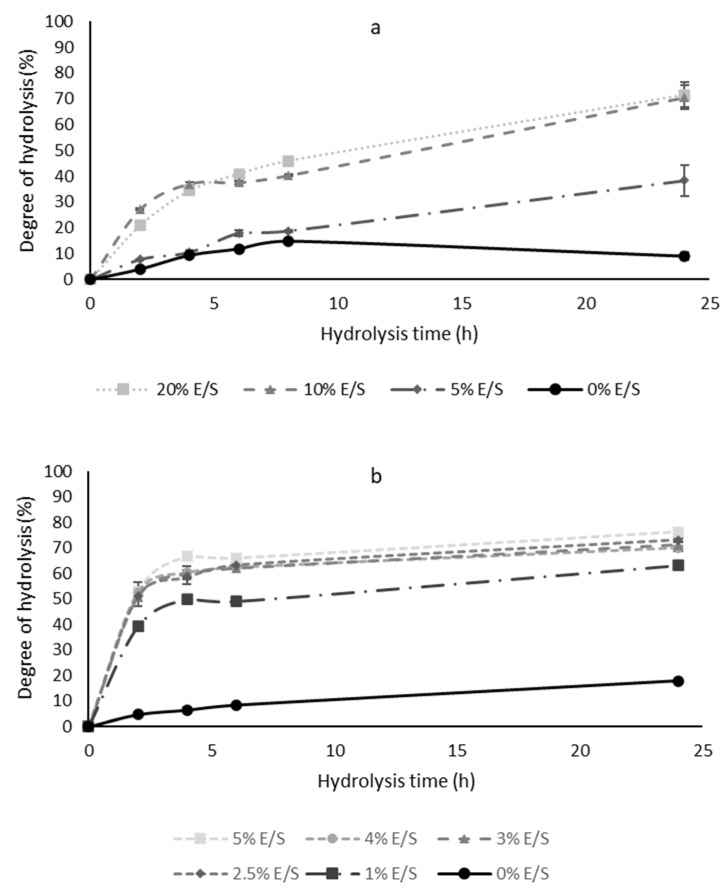
Degree of hydrolysis obtained from fish by-products with (**a**) viscera enzymatic extract and (**b**) alcalase.

The enzymatic hydrolysis of fish by-products was conducted at same optimum activity conditions of temperature and pH for viscera enzymatic extract and alcalase, i.e., 50 °C and pH 8.0 [25] for 24 h. Afterward, the process efficiency was measured by determining the DH (Figure 1), which is defined as the quantity of peptide bonds cleaved out of the quantity of peptide bonds in native protein [26]. As shown in Figure 1a, the viscera enzymatic extract added at amounts of 10 and 20% (E/S) exhibited similar DH profiles during all hydrolysis time. As observed, the DH reached approximately 40% at 6 h. The MW distribution of these FPH were also similar (Figure S1), revealing the production of peptides of low MW (below 13.7 kDa). Some studies have also produced FPH by treatment with a crude enzyme extract from fish viscera. Sayari and collaborators [27] obtained smooth hound (*Mustelus mustelus*) protein hydrolysates using intestine crude extract and gastric crude extract from the same species, which exhibited DH of 18.2% and 16.5% after 4 h, respectively. Those results indicated that intestine proteases had higher proteolytic activity compared to acidic proteases. Murphy and coworkers [28] also used a crude enzyme extract from tuna viscera (4.36% E/S) in combination with a commercial papain (0.81% E/S) for 60 min to prepare croaker protein hydrolysates with a DH of 38.75%.

Enzymatic hydrolysis of fish protein with commercial enzymes has become a widely used biotechnological process. As observed in Figure 1b, alcalase is capable of hydrolysing fish protein. The increase of enzyme concentration from 1% to 2.5% led to an increase of the DH; this result was similar to those reported by Altınelataman et al. (2019) [9]. On the other hand, alcalase used at amounts between 2.5 and 5% (E/S) displayed identical behaviour of hydrolysis, obtaining similar DH throughout the entire process. After 2 h of hydrolysis, DH values around 50% were reached. The action of the hydrolysis showed a high rate for the first 2 h and a slower rate until reaching a steady state. This behaviour of the DH curves is typical of the enzymatic hydrolysis when considering the quick cleavage of peptide bonds through the initial phase of hydrolysis, followed by a stationary stage where cleavage is reduced because of being less susceptible to the remaining bonds [29]. As DH curves were similar in a range of 2.5–5% E/S, the MW distribution of FPH was assessed (Figure S2), verifying the production of low MW peptides (below 13.7 kDa) using only 3% E/S of alcalase for 2 h. Alcalase has shown the ability to hydrolyse different fish species. *Micropogonias furnieri* and *Paralonchurus brasiliensis* muscle and skin tissues treated with the enzyme Alcalase 2.4 L^®^ had a DH of 40.9% and 42.8%, respectively [29]. European seabass (*Dicentrarchus labrax*) and gilthead seabream (*Sparus aurata*) muscles hydrolysates with alcalase showed a DH around 20% [9].

The extracts developed in the present study are considered highly hydrolysed. A high DH displays that a high peptide content was attained during the hydrolysis process, which increases the recovery of the protein. Thus, the selected conditions for the hydrolysis of fish by-products using the viscera enzymatic extracts were 10% (E/S) for 6 h and, using alcalase, were 3% (E/S) for 2 h.

Then, the characterization of FPH was performed; namely, nutritional, bioactive and technological properties.

### 2.4. Characterization of Fish Protein Hydrolysates

#### 2.4.1. Protein and Free Amino Acid Content

Fish protein hydrolysates had a protein content of 78.8 ± 3.5% (Dry weight basis—DW) and 83.6 ± 2.8 (DW) obtained with viscera enzymatic extract and alcalase, respectively. Several authors have found that the protein content of FPH ranges from 60% to 90% of the total composition [30,31,32]. The high protein content of the fraction is the result of the solubilization of proteins during the hydrolysis and the removal of insoluble solid matter by centrifugation [33].

The free amino acids compositions of FPH are shown in Table 3. The major amino acids present in both FPH were arginine, alanine, leucine and valine. Alanine is an important amino acid in muscle synthesis and is widely used as a supplement to athletic performance [34]. Dietary supplementation of arginine is a strategy that has the potential to be used in the treatment of diabetes [35] and as an immunological stimulator [36]. Alanine and arginine are stable amino acids, which are not destroyed by high temperatures and pressures. Valine, isoleucine, leucine (essential amino acids) and tyrosine (conditionally essential amino acid) play important roles in the synthesis of specific neurotransmitters, protein degradation and renewal, lymphocyte growth and proliferation, dendritic cell maturation, glycogen synthesis, and energy metabolism, among others. These amino acids have been reported as early indicators of cardiovascular problems, pancreatic adenocarcinoma, kidney disease and stroke [37].

The FPH obtained with viscera enzymatic extract also presented a higher amount of aspartic acid and glutamic acid. Aspartic acid is responsible for various biological activities in humans and animals. This amino acid is crucial for the chelation process of minerals (such as calcium and potassium, among others), improving their assimilation, digestion and utilization [38]. It is relevant for the synthesis of artificial sweeteners, such as aspartame [39]. Glutamic acid and glutamine are amino acids that are not synthesized by the body, but food industries use their flavour enhancement properties. For this reason, they are widely used, particularly in the form of monosodium salt.

The diversity of free amino acids existing in the FPH are recognized to have an influence on flavour and also to have a crucial role from a nutritional, chemical and biochemical perspective.

#### 2.4.2. Molecular Weight Distribution

**Figure 2:**
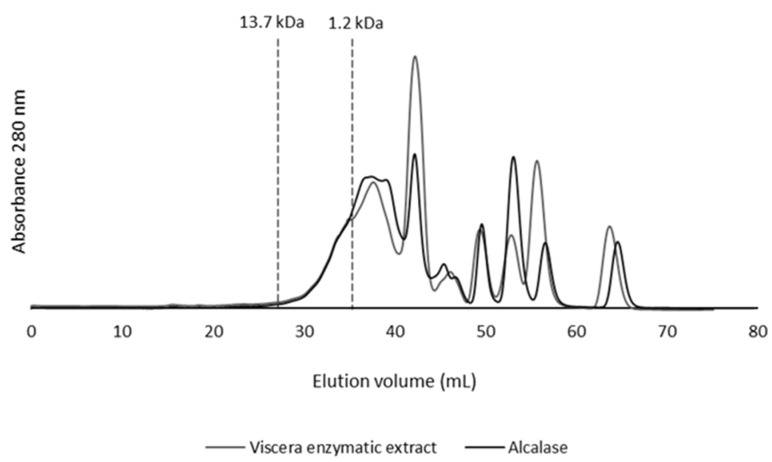
Molecular weight distribution of fish protein hydrolysates produced by enzymatic hydrolysis with viscera enzymatic extract and alcalase. Molecular weight markers of 13.7 kDa and 1.2 kDa are indicated.

The fish by-products subjected to hydrolysis with viscera enzymatic extract and alcalase were evaluated for their molecular weights (Figure 2); this allows understanding of the size and range of distribution of peptides developed in these properties. The chromatographic profiles revealed that FPH contain peptides with MW below 13.7 kDa, with a high contribution of peptides with MW smaller than 1.2 kDa. This result corroborated that protein in fish by-products was broken into low MW peptides or free amino acids by enzymatic hydrolysis. A high proportion of peptides below 6.5 kDa has been reported for FPH with alcalase via a high DH [15,18]. The existence of low MW peptides has also been directly associated with bioactive properties [18], enabling the use of PH as functional ingredients.

#### 2.4.3. Peptide Profile

**Figure 3:**
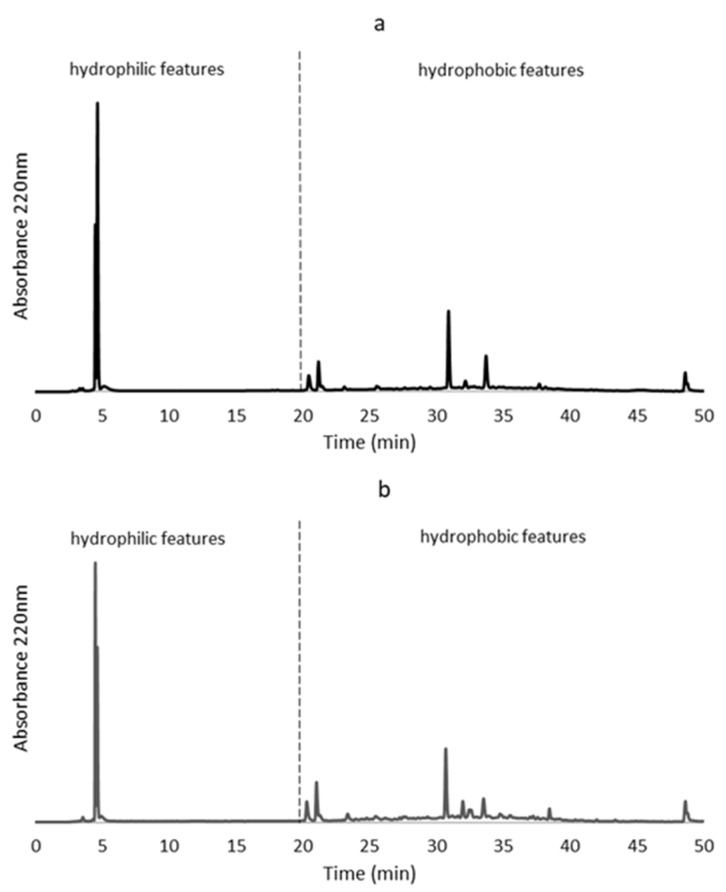
RP-HPLC chromatograms of fish protein hydrolysates obtained with (**a**) viscera enzymatic extract and (**b**) alcalase.

Fish protein hydrolysates were analysed by RP-HPLC, which allows understanding of the peptide composition by the adsorption analysis of peptides to a hydrophobic stationary matrix. The peptide profiles of both FPH are presented in Figure 3. The chromatograms of FPH obtained with viscera enzymatic extract and alcalase were similar. They showed a chromatographic peak between 0 and 20 min, which corresponds to peptides with hydrophilic characteristics. On the other hand, chromatograms showed a major elution of molecules between 20 and 40 min, corresponding to a phase more nonpolar, leading to the appearance of peptides with hydrophobic features. It is reported that peptides with antioxidant and ACE-inhibitory activities are commonly rich in hydrophobic amino acids/peptides, which improve absorption and interaction with free radicals or target enzymes [40].

### 2.5. Bioactive Properties of Fish Protein Hydrolysates

Bioactive properties of FPH were evaluated, regarding the antioxidant activity (by ABTS and ORAC methods) and antihypertensive activity (through the ability to inhibit the ACE). The stability of the bioactive properties of FPH was also tested, following in vitro simulation of the gastrointestinal tract (GIT) (Table 4). For this purpose, the conditions of the mouth (with α-amylase), the stomach (with pepsin) and intestinal digestion (pancreatin and bile salts) were simulated.

#### 2.5.1. Antioxidant Activity and Effect of Gastrointestinal Digestion

The antioxidant activity of peptides can be influenced by various factors, such as the degree of hydrolysis, molecular weights of peptides, amino acid sequences, and hydrophobicity/hydrophilicity [41]. In general, hydrolysates showed a higher antioxidant capacity than purified peptides [40].

The ABTS scavenging method assesses the capacity of the antioxidant compound to reduce the ABTS radical cation [42], while the ORAC method appraises the protection capacity of an antioxidant compound to reduce this peroxyl radical. The ORAC method is mainly used in the food industry as a reference to measure antioxidant ability. In the present study, the FPH exhibited antioxidant activity against both ABTS radical cations and peroxyl radicals, with similar values among enzymes (*p* > 0.05) (Table 4). Although the enzyme type plays an important role in the bioactivity of the produced hydrolysate, the antioxidant results indicated that hydrolysates produced by different enzymes were not significantly different. These findings are consistent with Lima et al. (2019) [16], where the elimination of the ABTS radicals by FPH was not influenced by different proteases.

Some studies verified that enzymatic hydrolysis of fish by-products with alcalase produced hydrolysates with antioxidant potential [10,16]. It is also known that smaller weight fractions reveal greater antioxidant activity [9,43]; as previously seen, the hydrolysates obtained with viscera enzymatic extract and alcalase contain low MW peptides. The hydrophobic amino acids present in the peptides (such as cysteine, histidine, tyrosine, methionine, tryptophan) also affect the antioxidant capacity [40], corroborating our results because FPH contained hydrophobic amino acids/peptides.

In general, the values of the antioxidant activity of FPH remained unchanged after the simulation of GIT (*p* > 0.05) (Table 4), although a slight activity increase was noted in alcalase hydrolysates against the ABTS radical cation (*p* < 0.05). These results demonstrate a great resistance of antioxidant peptides to GI digestion. These observations are in agreement with other studies, where fish hydrolysates also maintained, or even increased, the antioxidant activity after the passage of GIT [10,16,44].

#### 2.5.2. Ability to Inhibit the Angiotensin Converting Enzyme and Effect of Gastrointestinal Digestion

The antihypertensive activity of the FPH was performed according to the in vitro method that measures the inhibitory effect of the ACE (Table 4). The FPH produced by alcalase showed a better ACE inhibitory activity (IC_50_ of 101.1 µg of protein/mL) than FPH obtained by viscera enzymatic extract (IC_50_ of 554.4 µg of protein/mL) (*p* < 0.05). A similar IC_50_ value of alcalase hydrolysates was reported previously for Pacific hake FPH (161 μg of peptides/mL) [44].

The GI digestion also affected the ACE inhibitory activity in a different way (*p* < 0.05) (Table 4). In viscera enzymatic extract hydrolysates, the activity increased; however, in alcalase hydrolysates, the inhibitory activity of ACE decreased. In the literature, hydrolysates and individual ACE-inhibitory peptides are classified as “true-drug type”, “pro-drug type”, or “substrate type” based on unchanged, increased, or decreased ACE-inhibitory activity after simulated GI digestion, respectively [45]. Therefore, viscera enzymatic extract hydrolysates act as “pro-drug type” inhibitors and alcalase hydrolysates act as “substrate type” inhibitors.

Various effects of simulated GI digestion on ACE-inhibitory activity have been described. The ACE-inhibitory potential of Pacific hake FPH did not change upon simulated GI digestion [44]. The IC50 value of FPH produced with Protamex was reduced from 165 μg of peptides/mL to 90 μg of peptides/mL upon simulated GI digestion, thereby showing significantly greater ACE inhibitory activity [45]. On the other hand, simulated GI digestion lowered the ACE-inhibitory activity of both 10 kDa and 1 kDa ultrafiltrates of FPH [45]. It could also be suggested that a decrease in the ACE inhibitory potential could be a result of hydrolysis of the ACE-inhibitory peptides into smaller peptides or free amino acids during GI digestion [44].

### 2.6. Technological Properties

The technological properties of hydrolysates were evaluated using methodologies that allow the perception of oil absorption capacity [46], emulsifying properties and stability of the emulsifying properties of FPH [47] (Table 5).

The OAC is one of the most important technological properties used in the food industry and it can influence the taste of the product [33,48]. The FHP produced by viscera enzymatic extract and alcalase revealed an OAC with similar values among enzymes (*p* > 0.05). However, hydrolysates from tilapia by-products exhibited higher OAC values than the hydrolysates of the present study; over 2.0 g oil/g sample [49]. The same authors concluded that the enzymatic hydrolysis decreased the OAC. During this treatment, the integrity of protein structures and the physical entrapment of the oil are adversely affected because proteins are broken into smaller fragments [49].

The ability of proteins to form stable emulsions is also important because of the interactions between proteins and lipids in food systems [33]. In the present study, all the hydrolysates showed emulsifying properties, with values of approximately 40%. This capability was stable to heat treatment, also exhibiting a good emulsifying stability. Other FPH also revealed stable emulsions, such as catfish roe hydrolysates [15] and tilapia by-product hydrolysates [49].

Protein hydrolysates stabilize an oil-in-water emulsion due the hydrophilic and hydrophobic groups and charge. Hydrophobic peptides can be easily absorbed into the interfacial layers, while hydrophilic peptides usually remain in the aqueous. During enzymatic hydrolysis, the amino acids which have hydrophilic or hydrophobic features are more exposed. Therefore, these amino acids are acting as surfactants and promote the stability of an oil-in-water emulsion system [15]. Therefore, peptides with high hydrophobicity also have a critical role in the stability of the emulsion [40], which is related to the characteristics of the obtained FHP.

## 3. Materials and Methods

### 3.1. Materials and Reagents

Fish by-products from different species were obtained from ETSA (Loures, Portugal), a company specialising in the collection of animal by-products, and then viscera were separated. All reagents were purchased from Sigma-Aldrich (St. Louis, MO, USA) unless mentioned otherwise.

### 3.2. Composition Analysis of Fish By-Products and Viscera

The composition analysis of fish by-products (meat and bones) and viscera was performed according to the Association of Official Analytical Chemists’ procedures [50]. The protein content was determined by the Kjeldahl method and the nitrogen to protein conversion factor used was 6.25. The lipid content was determined gravimetrically after Soxhlet extraction of dried samples with hexane. The moisture was determined at 105 °C for 24 h. The ash content was determined at 550 °C for 5 h.

### 3.3. Preparation of Enzymatic Extract from Fish Viscera

Viscera of different fish species were obtained from a batch in substantial amounts to separate into two replicates. Viscera were homogenized with an extraction buffer of 10 mM Tris-HCl (pH 8) and 10 mM CaCl_2_ at a ratio of viscera:buffer solution of 1:2 (*w*/*v*). The crushing of viscera was performed with an ultraturrax (15,000 rpm, 1 min) in order to increase the amount of enzymes released, and then the mixture was homogenized in an orbital-shaker incubator (150 rpm, 10 min, 30 °C). The mixture was centrifuged at 8000 rpm for 15 min and the supernatant was collected and stored at −20 °C until use as crude protease extract.

#### 3.3.1. Determination of Proteolytic Activity of Viscera Enzymatic Extract

Protease activity of crude protease extracts and alcalase was measured using azocasein as a subtract, according to Alencar et al. (2003) [20] with some modifications. A 60 µL aliquot of the sample, suitably diluted, was mixed with 100 µL 200 mM Tris–HCl (pH 8.0) containing 1% azocasein and incubated for 60 min at room temperature (around 20 °C). The reaction was stopped by the addition of 480 µL 10% (*w*/*v*) trichloroacetic acid and centrifuged at 8000 rpm for 5 min to remove the precipitate. Then, 320 µL of the supernatant was added to 560 µL of 1 M NaOH and the absorbance measured at 440 nm against a blank prepared with the extraction buffer. One unit (U) of enzyme activity was defined as the amount of enzyme able to hydrolyse azocasein, giving an increase of 0.001 units of absorbance per minute. The measurements were analysed in duplicate for crude proteases extracts obtained with different ratios of viscera:buffer solution.

#### 3.3.2. Effect of pH on Activity of Viscera Enzymatic Extract

Protease activity was analysed in the pH range of 3–9 and measured using azocasein as a substrate [20]. For the measurement of the effect of pH on protease activity, the crude protease extracts were incubated for 1 h in different buffer solutions (sodium citrate at pH values of 3 and 5 and potassium phosphate at pH values of 6, 8 and 9) at room temperature (around 20 °C).

#### 3.3.3. Optimal Temperature of Viscera Enzymatic Extract

Optimal temperature was studied by incubating the crude protease extracts at 40, 50 and 60 °C at pH 8.0 and measured using azocasein as a substrate [20]. As the hydrolysis enzymatic is generally accomplished for prolonged periods of time, the optimal temperature of the enzymatic extract was evaluated for 4 h [21].

### 3.4. Enzymatic Hydrolysis of Fish By-Products

Fish by-products (meat and bones) were first minced in a cutting mill (CM 100 model, Porto, Portugal) and proteolysis was carried out using viscera enzymatic extract (catalytic activity of approximately of 70 U/mL) and also a commercial enzyme, alcalase (NewEnzymes, Maia, Portugal) (catalytic activity of approximately of 2600 U/mL). Several enzyme/substrate (E/S) ratios were tested to select the best condition of hydrolysis; viscera enzymatic extract was tested at ratios of E/S of 5, 10 and 20% (*v*/*w*) and alcalase was tested at ratios of E/S of 1, 2.5, 3, 4 and 5% (*v*/*w*). The enzymatic hydrolysis of fish by-products was conducted at optimal conditions previously established for viscera enzymatic extract and alcalase, i.e., 50 °C and pH 8.0. Aliquots were taken after 0, 2, 4, 6, 8 and 24 h. Then, the enzymes were inactivated by heat treatment at 95 °C for 15 min. The samples were centrifuged (8000 rpm, 10 min, 4 °C) and the supernatant was collected and stored at −80 °C for further analysis. Hydrolysis was carried out in duplicate for each assay and a control without the addition of enzymes was also tested.

Afterward, the degree hydrolysis (DH) and the peptide profile of the several protein hydrolysates obtained were analysed, in order to establish the best enzymatic hydrolysis conditions.

#### 3.4.1. Determination of Degree of Hydrolysis

The DH was evaluated by measuring the free amino groups by reaction of 2,4,6-trinitrobenzenesulfonic acid solution (TNBS), using the methodology described by Sousa et al. [43]. A reaction mixture with 50 μL of sample, 125 μL of 200 mM sodium phosphate buffer (pH 8.2) and 50 μL of TNBS at 0.025% were placed in a 96-well microplate (Sarstedt, Nümbrecht, Germany). The microplate was incubated at 45 °C for 1 h and the absorbance was measured at 340 nm. L-leucine (0.078–2.5 mM) was used to produce a standard curve. The DH was evaluated in duplicate for each condition. Then, DH was determined by the following formula:

DH (%) = (Lt − L0)/(Lmax − L0)*100

Lt is the quantity of amino groups released after hydrolysis time equal to t, L0 is the quantity of amino groups in the sample at initial hydrolysis time (blank) and Lmax is the maximum quantity of amino groups present in fish by-products. The Lmax was obtained by acid hydrolysis of fish by-products with 6 M HCl at 105 °C for 24 h. Then, the acid-hydrolysed sample was filtered and the supernatant was neutralized with 6 M NaOH before amino group acids assessment.

#### 3.4.2. Molecular Weight Distribution

Molecular weight (MW) distribution of FPH was determined by gel filtration chromatography using an FPLC system (AKTA pure, GE Healthcare Life Sciences, Chicago, IL, USA) coupled with two gel filtration columns: Superdex 200 increase10/300 GL and Superdex peptide, 10/300 GL. The eluent used was 0.025 M phosphate buffer (pH 7) containing 0.2 g/L of sodium azide and 8% NaCl at a flow rate of 0.5 mL/min. Elution was monitored at 280 nm and Thyroglobulin (669 kDa), Ferritin (440 kDa), Aldolase (158 kDa), Conalbumin (75 kDa), Ovalbumin (44 kDa), Carbonic anhydrase (29 kDa), Ribonuclease A (14 kDa) and Whey peptide (1 kDa) were used to perform the molecular weight standard curve. The results were expressed in milli Absorbance Units (mAU) per eluted volume (mL) [43].

### 3.5. Enzymatic Hydrolysis under the Best Conditions

After the enzymatic hydrolysis of fish by-products using viscera enzymatic extract and alcalase, the optimal condition was defined based on the degree of hydrolysis and MW distribution analysis. The selected conditions for the hydrolysis using the viscera enzymatic extract were 10% (E/S) for 6 h and using alcalase for 3% (E/S) at 6 h. The enzymatic hydrolysis with the selected conditions was performed as described in Section 2.3. The supernatant, rich in protein hydrolysates, was freeze dried (Armfield SB4 model, Hampshire, England) and stored in a desiccator for further characterization.

### 3.6. Characterization of Fish Protein Hydrolysates

#### 3.6.1. Protein and Free Amino Acid Content

The protein content was determined by the Kjeldahl method and the nitrogen to protein conversion factor used was 6.25 [50].

Free amino acids content of FPH was made by pre-column derivatization with orthophthalaldehyde (OPA) methodology. Isoindole-type fluorescent derivatives were formed in an alkaline solution (borate buffer pH 10.4) from OPA, 2-sulfanylethanol and the primary amine group of the amino acid. The derivatives were separated by reverse phase-high performance liquid chromatography (HPLC) (Beckman coulter, USA), coupled to a fluorescence detector (Waters, Milford, MA, USA), according to Proestos et al. (2008) [51]. Each sample (100 μL) was derivatized and the injection volume of derivatives was 20 μL. All analyses were performed in duplicate and quantified using a calibration curve created with amino acids pure standards and expressed as mg/g of protein.

#### 3.6.2. Molecular Weight Distribution

The MW distribution of FPH was achieved by a gel filtration chromatography as described in Section 3.4.2.

#### 3.6.3. Peptide Profile

Peptide profiles of FPH were analysed by RP-HPLC using a Beckman Coulter unit equipped with Karat32 software and a C18 column (COSMOSIL 5C18-AR-II), maintained at room temperature. Separation was made using two eluents: 0.1% trifluoroacetic acid (TFA) in ultrapure water (*v*/*v*) (eluent A), and 0.1% TFA in acetonitrile (*v*/*v*) (eluent B). The gradient elution was as follows: 0–20 min 0% of B; 20–40 min: gradient B increase until 100% and finally 40–50 min B decrease until 0%. The flow rate was 0.8 mL/min and the detection was achieved at 220 nm (Beckman Diode Array 168, East Lyme, CT, USA). The volume injected was 20 μL and the analysis was performed in duplicate.

### 3.7. Bioactive Properties of Fish Hydrolysates

#### 3.7.1. Analysis of Antioxidant Activity

##### ABTS Scavenging Assay

The ability of free radical-scavenging by FPH was evaluated through 2,2-azino-bis-3-ethylbenzothiazoline-6-sulphonic acid (ABTS) radical decolourization assay [42]. The radical cation was generated by reacting ABTS with potassium persulfate. Then, 1 mL of ABTS solution was reacted with the sample for 6 min and then the absorbance was measured at 734 nm. A standard curve was carried out with ascorbic acid in the range of 0.063–0.250 mg/mL. All the measurements were performed in triplicate, and results were expressed as mg ascorbic acid equivalent/g dry extract.

##### ORAC Assay

The measurement of oxygen radical absorbance capacity (ORAC-FL) was performed according to Ou et al. 2021 [52]. Samples were dissolved in 75 mM phosphate buffer (pH 7.4) and the solution was placed in a black 96-well microplate (Nunc, Roskilde, Denmark), mixed with 120 μL of fluorescein (70 nM) and incubated at 40 °C for 10 min. Then, 60 μL of 2,2′-Azobis(2-amidinopropane) dihydrochloride (AAPH) solution (14 mM) was added to the mixture. The fluorescence was recorded for 140 min using a microplate reader (Synergy H1, Santa Clara, CA, USA), at excitation and emission wavelengths of 485 and 528 nm, respectively. The area under curve (AUC) was calculated for each sample by integrating the relative fluorescence curve. Trolox (9.98 × 10^−4^–7.99 × 10^−3^ μmol/mL) was used as the standard, then regression equations for Trolox and samples were calculated. All reactions were performed in duplicate, and three independent runs were carried out. Final ORAC values were expressed as mg Trolox equivalent/g dry extract.

#### 3.7.2. Measurement of Angiotensin Converting Enzyme (ACE) Inhibitory Effect

The inhibitory effect of Angiotensin-converting enzyme (ACE) was measured as described by Sousa et al. (2020) [43]. The o-Abz-Gly-p-Phe(NO2)-Pro-OH (0.45 mM) (Bachem, Bubendorf, Switzerland) was used as a substrate, and the reaction performed in 0.04 U/mL of ACE (peptidyl-dipeptidase A, EC 3.4.15.1) pH 8.3 with 0.1 mM ZnCl2. The reaction mixture was incubated at 37 °C and the fluorescence was recorded after 45 min using a FLUOstar OPTIMA plate reader. The wavelengths used were 350 nm (excitation) and 420 nm (emission). Non-linear fitting to the data was performed to calculate the IC50 (protein concentration needed to inhibit 50% of ACE activity) [53]. The ACE inhibitory activity was analysed in duplicate for each sample.

### 3.8. Simulation of Gastrointestinal Tract Conditions

The bioactive properties of FPH obtained under the best conditions were also evaluated after passage throughout the simulated gastrointestinal system, as described by Amorim et al. (2019) [54]. Mouth digestion was conducted with 0.6 mL of α–amylase solution (100 U/mL) and incubation occurred for 1 min at 37 °C and 200 rpm. For gastric digestion, pH was adjusted to 2.0 with concentrated HCl (6M) and the mixture was incubated with 25 mg/mL of pepsin (from porcine stomach mucosa, pepsin A 250 U/mg), at a rate of 0.05 mL/mL−1 of sample for 60 min at 37 °C and 130 rpm. For intestinal digestion, the pH was adjusted to 6.0 with NaHCO_3_ (1 M), then pancreatin 2 g/L (from porcine pancreas 8 x USP), and 12 g/L of bile salts were added at a ratio of 0.25 mL/mL of sample and incubation occurred for 120 min at 37 °C and 45 rpm. This assay was carried out in duplicate. After digestion, antioxidant activity and ACE inhibitory effect were measured according to the methodologies described above.

### 3.9. Technological Properties

#### 3.9.1. Oil Absorption Capacity

The oil absorption capacity (OAC) was evaluated according to Isah et al. (2017) [46], with some modifications. Samples were added to sunflower oil at a ratio of 1:10 (*w*/*v*) and then heated in a water bath at 60 °C for 30 min. The mixture was centrifuged at 1000 rpm for 15 min. The supernatant was carefully decanted and weighed. The OAC was expressed as grams of oil retained per gram of sample.

#### 3.9.2. Emulsifying Capacity and Stability

The emulsifying capacity (EC) and stability were determined using the Chaparro et al. (2012) methodology [47] with some modifications. Samples were dispersed in distilled water (10 mg/mL); therefore, the protein proportion in FPH produced with viscera enzymatic extract and alcalase used in this assay was approximately 7.88 mg and 8.36 mg, respectively. Then, samples were homogenized with sunflower oil at a ratio of 1:1 (*v*/*v*). The emulsions were centrifuged at 1100 rpm for 5 min. The height (cm) of the emulsified layer (ELH) and height of the total content of the tube (TC) were registered. The EC was calculated as:

EC (%) = ELH/(TC)*100

Emulsion stability (ES) was evaluated by heating the emulsion at 80 °C for 30 min before centrifuging at 1100 rpm for 5 min. The height (cm) of the emulsified layer after heating (ELHA) and the height (cm) of total content before heating (TCA) were measured. The ES was calculated as:

ES (%) = ELHA/(TCA)*100

### 3.10. Statistical Analysis

Data were analysed using *t*-test at a significance level of 0.05, using the Statistical Package for Social Sciences software (version 21, SPSS, Armonk, NY, USA). Results were expressed as means of at least two replicates.

## 4. Conclusions

The production of protein hydrolysates from fish by-products could be accomplished with the aid of 10% viscera enzymatic extract for 6 h, reaching DH close to the equivalent hydrolysis with 3% commercial alcalase for 2 h (40 and 50%, respectively). The FPH showed to be a valuable resource of protein and amino acids. Furthermore, the hydrolysis released peptides with antioxidant and ACE inhibitory activity, maintaining the properties after passing through the simulated GIT. The FPH also had oil absorption capacity and emulsifying properties. The biological and technological properties of FPH obtained by 10% viscera enzymatic extract were similar to that obtained by 3% alcalase, except for antihypertensive activity, which was significantly higher for alcalase.

The fish by-product protein hydrolysates are a promising source of compounds with bioactive properties. Therefore, they could be used as ingredients in functional food development and offer an alternative with higher value than the current simple production of fish-meal. In this way, the enzymes and the hydrolysates are obtained using the same by-products, being aligned with the concept of a circular economy, using a self-sufficient and zero waste approach and obtaining circular value-added products.

## Figures and Tables

**Table 1:** Proximal composition of fish by-products and viscera.

	Fish By-Products	Viscera
Moisture (%)	74.5 ± 0.6	66.9 ± 0.1
Protein (%)	17.7 ± 1.0	12.4 ± 0.6
Fat (%)	2.0 ± 0.0	18.6 ± 0.5
Ash (%)	3.9 ± 0.5	2.0 ± 0.1
Carbohydrate (%) *	1.9	0.1

* Carbohydrate calculated by difference.

**Table 2:** Effect of pH and temperature on the activity of viscera enzymatic extract.

pH Values	U/mL
3	5.64 ± 0.00 ^a^
5	17.36 ± 0.20 ^b^
6	30.78 ± 0.02 ^c^
8	67.72 ± 1.05 ^d^
9	68.83 ± 1.20 ^d^
**Temperature (°C)**	
40	77.16 ± 1.22 ^e^
50	71.65 ± 1.65 ^e^
60	27.45 ± 0.13 ^f^
**Alcalase**	2602.22 ± 80.30

Means with different superscripts within the same parameter are statistically different (*p* < 0.05).

**Table 3:** Free amino acid composition of fish protein hydrolysates.

Amino Acids (mg/g Protein)	Viscera Enzymatic Extract	Alcalase
Aspartic acid	7.84 ± 0.12	2.44 ± 0.01
Glutamic acid	9.49 ± 0.16	3.00 ± 0.04
Cysteine	0.11 ± 0.01	0.46 ± 0.03
Asparagine	0.24 ± 0.00	1.35 ± 0.02
Serine	3.66 ± 0.03	2.39 ± 0.05
Histidine *	1.65 ± 0.14	3.61 ± 0.16
Glutamine	3.41 ± 0.01	3.38 ± 0.05
Threonine *	5.64 ± 0.06	4.55 ± 0.08
Arginine	14.79 ± 0.11	6.14 ± 0.18
Alanine	12.45 ± 0.07	7.62 ± 0.13
Tyrosine	4.53 ± 0.08	2.82 ± 0,06
Valine *	7.74 ± 0.06	5.15 ± 0.09
Methionine *	5.20 ± 0.09	4.35 ± 0.02
Tryptophan *	0.98 ± 0.04	0.72 ± 0.03
Phenylalanine *	5.64 ± 0.40	4.64 ± 0.18
Isoleucine *	5.85 ± 0.06	3.38 ± 0.04
Leucine *	11.65 ± 0.06	9.66 ± 0.27
Total	100.86	65.66

* Essential amino acids.

**Table 4:** Antioxidant activity and ACE inhibition activity values of fish protein hydrolysates, before and after simulation of gastrointestinal digestion.

Fish Hydrolysates	ABTS (mg Ascorbic Acid/g Dry Extract)		ORAC (mg Trolox/g Dry Extract)		IC_50_ (µg of Protein/mL)	
	Before GIT	After GIT	Before GIT	After GIT	Before GIT	After GIT
Viscera enzymatic extract	10.4 ± 0.9 ^a,b^	10.7 ± 0.9 ^a,b^	142.0 ± 4.2 ^c^	148.3 ± 4.8 ^c^	554.4 ± 10.1 ^d^	430.6 ± 13.2 ^f^
Alcalase	11.0 ± 0.5 ^a^	13.7 ± 0.0 ^b^	153.2 ± 29.4 ^c^	127.4 ± 54.9 ^c^	101.1 ± 2.0 ^e^	155.3 ± 4.0 ^g^

Means with different superscripts within the same methodology are statistically different (*p* < 0.05).

**Table 5:** Technological properties of fish protein hydrolysates.

Fish Hydrolysates	Oil Absorption Capacity (g Oil/g Sample)	Emulsifying Property (%)	Stability of Emulsifying Property (%)
Viscera enzymatic extract	1.07 ± 0.08 ^a^	41.9 ± 0.3 ^b^	41.4 ± 0.4 ^b^
Alcalase	1.06 ± 0.17 ^a^	41.1 ± 1.5 ^b^	41.1 ± 1.5 ^b^

Same letters mean no statistically significant differences (*p* > 0.05).

## Data Availability

Data is contained within the article or Supplementary Materials.

## Supplementary Materials

The following supporting information can be downloaded at: https://www.mdpi.com/article/10.3390/molecules28031155/s1, Figure S1: Molecular weight distribution of fish protein hydrolysates produced by enzymatic hydrolysis using different amounts of viscera enzymatic extract: 20% (E/S) (); 10% (E/S) () and 0% (E/S) () after 6 h of hydrolysis; Figure S2: Molecular weight distribution of fish protein hydrolysates produced by enzymatic hydrolysis using different amounts of alcalase: 5% (E/S) (); 4% (E/S) (); 3% (E/S) (); 2.5% (E/S) () and 0% (E/S) () after 2 h of hydrolysis.

## Sample Availability

Not available.
