# Supplementary material for: Fish By-Products: A Source of Enzymes to Generate Circular Bioactive Hydrolysates
Source: Molecules. 2023 Jan 24;28(3):1155. doi: 10.3390/molecules28031155 (PMC9919145; doi:10.3390/molecules28031155)
Supplement: Supplementary file 1 [file molecules-28-01155-s001.zip › molecules-2106327-supplementary.pdf]

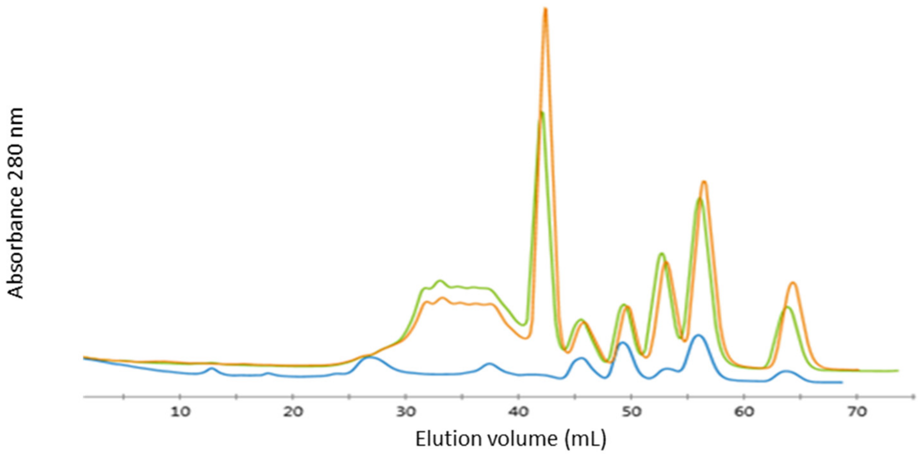

Figure S1: Molecular weight distribution of fish protein hydrolysates produced by enzymatic hydrolysis using different amounts of viscera enzymatic extract: 20% (E/S) (▬); 10% (E/S) (▬) and 0% (E/S) (▬) after 6 h of hydrolysis.

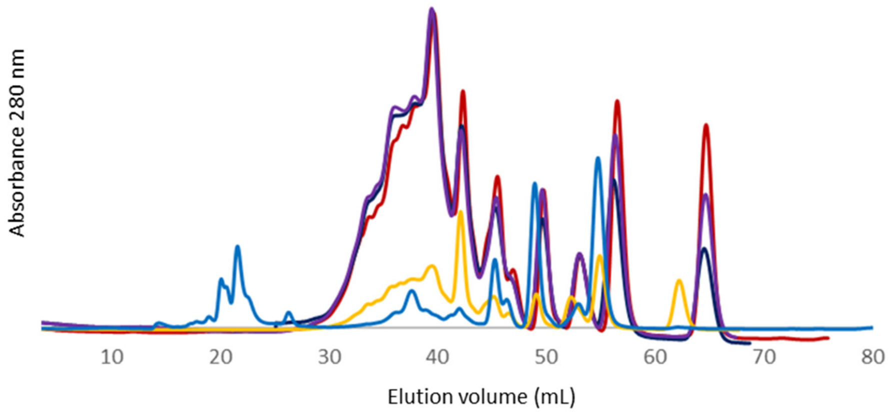

Figure S2: Molecular weight distribution of fish protein hydrolysates produced by enzymatic hydrolysis using different amounts of alcalase: 5% (E/S) (▬); 4% (E/S) (▬); 3% (E/S) (▬); 2.5% (E/S) (▬) and 0% (E/S) (▬) after 2 h of hydrolysis.
